# Leveraging Artificial Intelligence and Fleet Sensor Data towards a Higher Resolution Road Weather Model

**DOI:** 10.3390/s22072732

**Published:** 2022-04-02

**Authors:** Toon Bogaerts, Sylvain Watelet, Niko De Bruyne, Chris Thoen, Tom Coopman, Joris Van den Bergh, Maarten Reyniers, Dirck Seynaeve, Wim Casteels, Steven Latré, Peter Hellinckx

**Affiliations:** 1 IDLab-Faculty of Applied Engineering, University of Antwerp-IMEC, Sint-Pietersvliet 7, 2000 Antwerp, Belgium; toon.bogaerts@uantwerpen.be (T.B.); steven.latre@uantwerpen.be (S.L.); peter.hellinckx@uantwerpen.be (P.H.); 2 Royal Meteorological Institute of Belgium, Ringlaan 3, 1180 Brussels, Belgium; swatelet@meteo.be (S.W.); jorisvdb@meteo.be (J.V.d.B.); maarten.reyniers@meteo.be (M.R.); 3 Verhaert Masters in Innovation, Hogenakkerhoekstraat 21, 9150 Kruibeke, Belgium; niko.debruyne@verhaert.com (N.D.B.); chris.thoen@verhaert.com (C.T.); dirck.seynaeve@verhaert.com (D.S.); 4 Inuits-Open Source Innovators, Essensteenweg 31, 2930 Brasschaat, Belgium; tom.coopman@inuits.eu

**Keywords:** vehicle data, smart sensors, artificial intelligence, machine learning, road safety, road weather conditions, road weather models, road weather services, nowcasting, weather warnings

## Abstract

Road weather conditions such as ice, snow, or heavy rain can have a significant impact on driver safety. In this paper, we present an approach to continuously monitor the road conditions in real time by equipping a fleet of vehicles with sensors. Based on the observed conditions, a physical road weather model is used to forecast the conditions for the following hours. This can be used to deliver timely warnings to drivers about potentially dangerous road conditions. To optimally process the large data volumes, we show how artificial intelligence is used to (1) calibrate the sensor measurements and (2) to retrieve relevant weather information from camera images. The output of the road weather model is compared to forecasts at road weather station locations to validate the approach.

## 1. Introduction

Weather conditions have a significant impact on the accident risk on road networks [1]. These accidents can lead to traffic jams, increased pollution, material costs, and person injuries. Visibility on the road can be affected by fog or rain showers. The road surface is also affected by weather conditions; for example, snow or freezing rain can cause slippery road conditions. These types of conditions can cause dangerous situations for drivers. Furthermore, they can also impact advanced driver assistance systems [2]. The technologies designed for self-driving vehicles also have a varying performance under different weather conditions [3].

To forecast dangerous road weather conditions, various dedicated road weather models (RWM) have been developed in the past [4,5,6,7,8,9]. These models are usually run for specific locations with fixed road weather stations (RWS) that provide the necessary observations such as the road surface temperature. RWS do not always cover the entire the road network: when sparsely scattered, they might miss very local phenomena. RWMs can be integrated into intelligent transport systems to increase safety and tackle growing emission and congestion problems [10,11]. In this work, we explore the potential improvement that a vehicle fleet, equipped with sensors, can bring to road weather models. Previous research has shown potential with regard to the assimilation of mobile road surface temperature observations [12]. Various vendors have developed mobile sensors for this purpose [13]. Accurate forecasts of dangerous road conditions allow the distribution of real-time warnings to nearby vehicles to alert drivers or to inform road management authorities, who can take preventative measures such as salting roads or measures such as snow removal after an event. The Finnish Meteorological Institute recently introduced a system to distribute road weather data amongst vehicles [14], and in [15], the potential of floating car data was also addressed. The potential of vehicles-based observations is continuously increasing [16]. The value of the connected car market has tripled between 2012 and 2019 [17]. It is expected to grow further towards $212.7 billion by 2027 [18]. A fleet of smart connected vehicles could provide us with fine-grained observations, allowing the prediction of local road weather conditions and the generation of real-time warnings. Furthermore, these observations have the potential to improve numerical weather prediction (NWP) model forecasts [19,20].

A group of industrial stakeholders and researchers consisting of more than twenty partners from seven countries (Belgium, France, Portugal, Romania, Spain, Turkey, and South Korea) initiated the real-time location-aware road weather services composed from multi-modal data (SARWS) project [21]. The SARWS project aims to enable real-time weather services using data originating from large-scale vehicle fleets. This paper is written by the Belgian consortium, which aims to utilise vehicle-fleet observations for real-time local road weather condition detection.

Local measurements are gathered around the city of Antwerp in Belgium from a fleet of postal vans [22]. The vehicles are equipped with various external sensors. The collected data are first centralised and (pre-)processed in the car at the In Car Smart Sensor Node (ICSSN) before being further distributed to the cloud. To obtain relevant information from the large data volumes, machine learning techniques are leveraged. In this paper, we create a convolutional neural network (CNN) to obtain information about the visibility and precipitation from the camera images and explore how neural network can be used to calibrate the sensor measurements. We also demonstrate how the collected data can be used to enhance the road weather model of the Royal Meteorological Institute of Belgium (RMI) at a high resolution.

The next section presents the different materials and methods that are used and some corresponding state-of-the-art. The results are presented in Section 3 and finally, in Section 4, the conclusions are presented.

## 2. Materials and Methods

### 2.1. Detection of Precipitation Type and Visibility from Camera Images

As described in Section 1, the ICSSN contains several sensors, which cover different complementary types of perception, to enhance road weather services. To detect precipitation, and more specifically precipitation type (e.g., rain, snow, …), and visibility (fog), visual perception is considered and investigated as an important sensor. Our approach to this multi-task problem of detecting precipitation type and visibility is a smart sensor, WeathercAIm, consisting of a combination of a camera with edge artificial intelligence (AI) computer vision (CV) processing. This smart sensor is part of the ICSSN and processes the camera images locally.

Recent research on weather detection from images applies both classical CV approaches as—with the rise of deep neural networks (DNNs) and especially CNNs for CV tasks–DNN based methods [23,24]. DNN-based methods have the advantage of increasing the accuracy with the amount of data available. In recent years, street-level images in combination with DNN have shown the potential of AI for weather detection [23,24,25,26,27]. These related works are mainly theoretical, whereas we also deploy the WeathercAIm in an actual vehicle fleet and tackle the problem together with the more practical side and constraints.

From the practical side, one of the important aspects for the WeathercAIm design is where to deploy the AI model: in the cloud or locally on the ICSSN. Sending images frequently over the data (radio) network to be processed in the cloud would massively increase the network communication traffic. The bandwidth needed would drastically limit the scalability to large vehicle fleets. Moreover, to comply with the General Data Protection Regulation (GDPR), sending and storing data that could contain privacy sensitive information in the cloud should be avoided. With these constraints, i.e., optimising communication and keeping privacy in mind, the choice for edge AI where the images are immediately processed locally on the ICSSN is evident [28,29,30,31]. There is no need for the images to be stored, not even locally. The available computing resources, e.g., processing power and memory, on edge platforms are typically lower than in the cloud [29,30,32]. Thus, edge AI has its implications on the AI model that can be used as it puts tighter constraints on the computing resources. Larger or complex AI architectures, e.g., using multiple parallel or cascaded CNNs and/or combined with other DNNs as in [23,25,26], could make the solution too computationally heavy or memory hungry to fit on an edge platform. The inference—detection processing—time will also take longer for more complex AI architectures. Another specific of the SARWS project is that a prediction is desired for every road segment of 50–250 m. This puts an extra timing constraint on the inference time. Complex AI architectures will likely take too long to comply to these constraints on an edge platform.

The proposed design of WeathercAIm is a camera connected to the ICSSN. The ICSSN also runs the precipitation type and visibility AI model besides running and processing all other vehicle sensors. The camera is a Basler puA1920-30uc [33]. This is a low-cost compact and low-weight area scan camera. The camera is placed behind the windshield close to the rear-view mirror. For the machine learning (ML) model, a supervised learning approach is used for multiclass classification, using a classifier based on a CNN that has proven its merits in the computer vision field. We use a single CNN handling both precipitation type and visibility detection. The current implementation is the well-known, seasoned ResNet50v2 [34] backbone and a dense layer as the classification head. This was chosen as it was considered a reference for image classification with a good balance between learning capabilities, accuracy, needed compute resources, and inference time. It has been studied extensively in the literature [32,35] and shown to be working and even used as a reference benchmark on edge platforms [31,36,37]. So, it was likely to also comply to the constraints discussed above and deployable in the field on the ICSSN edge platform, which made it a sensible choice. The CNN is trained via transfer learning, with the backbone network pre-trained on ImageNet [38]. Cross-entropy is used as loss function, and different optimisers (stochastic gradient descent (SGD) and Nadam) are evaluated. To counter overfitting, image augmentation, i.e., rotation, horizontal flip, translation, and zoom, regularisation and dropout layers are used. The CNN is implemented in Keras/TensorFlow. For the inference and deployment on the actual target/ICSSN, TensorFlow was compiled from source to be able to run on and use the target specific processor (Intel Atom x7-E3950) optimisations for optimal performance.

To train and validate ML models, having enough good data, and in the case of supervised learning good, labelled data, is vital. The better the dataset represents the data in production, the better the performance of the ML model. The data weather classes/labels of interest to enhance the road weather services and which need to be represented in the dataset are:

- Precipitation: rain, melting snow, snow, hail, no precipitation;
- Visibility: fog, normal (no fog)

**Figure 1:**
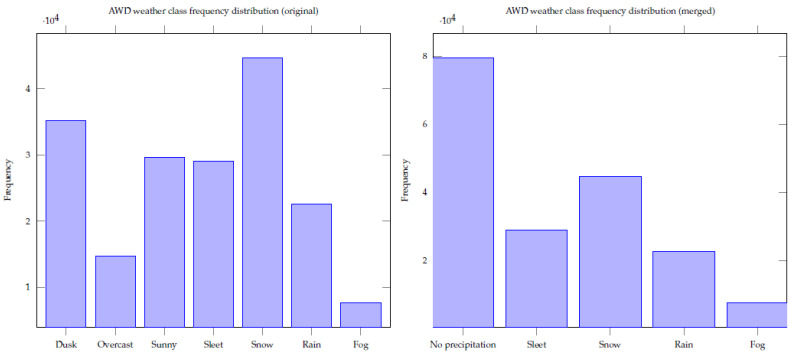
AWD weather class frequency distributions.

To gather a representative dataset, images were collected during the beginning of the data-gathering campaign, the so-called development phase, with the first 3 ICSSN equipped vehicles between 23 October and 31 May 2021. We also performed a dataset survey to search for relevant publicly available weather-related datasets to build an initial model. Finally, the “Adverse Weather Dataset” (AWD) [39] was used for the first validation of the approach as it covers most dataset requirements (except for the lack of hail). In addition to the weather classes of interest, the dataset also contains other classes such as dusk, overcast, and sunny. These were merged to the weather classes of interest; e.g., if classified as just sunny, this corresponds to no precipitation, see the AWD weather class frequency distribution plots in Figure 1.

Although the AWD classes seem quite balanced, in reality, the occurrence of no precipitation is much higher than the precipitation types (especially the rarer types such as hail or fog). So, we decided to keep and split the no-precipitation class into subclasses, e.g., overcast, sunny, to avoid class imbalance issues during modelling. A simple rule-based system is used after the CNN to map these extra no-precipitation classes to the classes of interest.

The results presented in Section 3.1 are from the initial model experiments on the public AWD dataset.

### 2.2. Calibration of Sensor Measurements

The need for the calibration of low-cost measurements is presented by Williams et al. [40]. The authors state that regular calibration can improve data believability, but manual calibration can be costly. Previous research has been done to calibrate mobile sensors using machine learning regarding urban air quality [41]. Furthermore, mobile sensor calibration using sensor fusion and mixed models for road weather predictions has proven its value, as shown by Lovén et al. [42]. In this paper, we investigate a complete data-driven calibration of our low-cost sensor measurements. We are currently collecting observations from 15 postal vans. These measurements are environmental observations such as the ambient temperature. Every time the Global Positioning System (GPS) is updated (on average 2–3 s), the sensor measurements are collected and sent to the cloud. These sensors provide the input for our applications. Table 1 gives an overview of the external sensors. The camera is mounted behind the windscreen together with the GPS, the thermal image sensor is mounted at the front of the vehicle and aimed at the road surface, and other sensors are placed in a sensor box on top of the vehicle. These measurements are validated by comparing the observations by our mobile sensors to meteorological forecasts. This is a continuation of our previous research [22,43]. Given the large amounts of generated data, artificial intelligence and machine learning are perfectly suited to improve the sensor accuracy [15]. The accuracy of data-driven approaches rises with the amount of data available, outperforming statistical methods. For this, we use a fully connected neural network. These networks take a certain number of inputs and convert them with trainable parameters to construct the output.

As discussed later in Section 3.3, the measurements of the sensors differ from the RWS. For example, this can be caused by sensor placement or vehicle speed. Our mobile sensing fleet covers a large area. However, we cannot calibrate using only fixed weather stations, as they are sparsely spread. We do have the output from weather forecasts at a high resolution of 10 min and on a 1 km × 1 km grid. We use these outputs as pseudo labels for our calibration model. These outputs might not be the real ground truth but are expected to indicate trends where our measurements are incorrect. As we have a lot of data, we explore the possibilities of data-driven calibration. Our model consists of three fully connected layers with 10 neurons, 64 neurons, and 1 neuron. The first two layers have a Rectified Linear Unit activation function to allow for sparse activation, which can be useful to give less attention to outliers. The input of the model is the measured T2M, RH, and GPS velocity. Two models are created; the output is either the forecasted T2M or RH. Our model is trained using the Adam optimiser and the mean absolute error as loss function. The model is trained for 20 epochs and a batch size of 500.

### 2.3. Road Weather Model

The Royal Netherlands Meteorological Institute developed an RWM [44] that was used as a basis for the RWM of the RMI. Within this model, further referred to as the “classic RWM”, the road is modeled up to a depth of 30 cm and is divided into 20 asphalt layers. The RWM computes the energy balance at the surface of the road where incoming and outgoing radiation, atmospheric, and ground heat fluxes are considered. Changes of state (evaporation, melting, etc.) are also taken into account. Several parameters can be fine-tuned to better match the modeled RST with observations, with the possibility of factoring in environmental conditions such as a sky-view factor and the presence of bridges [44]. The initial conditions are updated by performing a data assimilation of RWS observed road surface temperature (RST). The observed air temperature (T2M) and dew point temperature (TD2M) are also used to correct the NWP forecast at the RWS location.

In order to use the SARWS data to their full potential, we developed an enhanced version of the classic RWM, named after the project: RWM_SARWS. This enhanced RWM not only uses weather observations from the Flemish Road and Traffic Agency RWS but also the near real-time SARWS car sensor data aggregated at the road segment level (∼50 m). These observations are available for the region of Antwerp and are aggregated by Be-Mobile. In addition to the observations, RWM_SARWS uses weather input from an NWP model, which can be selected by the RMI forecasters based on the expected accuracy in given meteorological conditions. This NWP model output is combined with the output of RMI’s operational nowcasting system INCA-BE (Integrated Nowcasting through Comprehensive Analysis Belgium). INCA-BE is an adapted and extended version of the INCA system [45] developed at the national meteorological service of Austria. It generates high-resolution short-term forecasts integrating observational data from weather radars, synoptic stations, and other official networks. Due to the importance of precipitation nowcasts for road weather warnings, we ingest the INCA-BE precipitation information at a high resolution of 10 min in RWM_SARWS.

Every 20 min, RWM_SARWS forecasts are performed for each road segment in Antwerp and its surroundings, which corresponds approximately to 140,000 road segments. Each of these has a length of about 50 m. Since the SARWS project focuses on nowcasting, the forecast range is limited to two hours. RST, T2M, TD2M, and road surface condition as well as the amounts of liquid water, ice, and snow on the road are provided as output.

RWM_SARWS makes use of the observed RST, T2M, and TD2M from RWS and car sensors, with the same correction methods employed as for the classic RWM. A new version of RWM_SARWS is currently under development that will make use of car wiper information and the predictions from the WeathercAIm. When rain is deduced from the car sensor observations, the RWM road surface status can be updated to wet, which can then be taken into account in the forecast for that road segment. This information can be validated when the car is close to the RWS, which measures road surface status and rain state (rain occurring yes/no).

## 3. Results

### 3.1. Detection of Precipitation Type and Visibility from Camera Images

We present our experiments to validate our approach by training and testing an initial WeathercAIm model with the Adverse Weather Dataset [39] before deployment. The considered weather classes are fog, dusk, overcast, rain, sleet, snow, and sunny. The dataset was split in a training and validation set according to an approximate 80/20 grouped stratified split on drive sequence for each weather class. Except for fog, which was present during only one drive sequence, which was then split taking the first 80% samples for training and the last 20% for validation. The experiments were run with different hyperparameters for the CNN to train and tune the network: learning rate, dropout rate, batch size, and whether the pre-trained backbone layers should be fixed or fine-tuned. A selection of the results are presented in Table 2 for multiclass weather classification on the 7 classes defined in the dataset with different performance validation metrics (accuracy, area under the ROC curve (AUC), and F1 score), for different hyperparameters.

Using data augmentation, a pre-trained backbone with fine tuning and SGD (with momentum 0.9) as optimiser gave the best results in our experiments. These results reveal that the WeathercAIm CNN can learn the weather classes with a good accuracy based on the given dataset.

The model was also deployed on the ICSSN in vehicles in the field. Initial results from the field test development phase—running from 23 October 2020 to 31 May 2021—reveal that there is still room for improvement in accuracy on real-life production data. One of the main observations is that the resolution of the camera does not allow for a high performance in changing lighting conditions, producing lesser quality images than the camera images from the AWD dataset:

- Automatic gain functions take time to settle on initial captures and on big lighting changes e.g., coming out of a tunnel.
- Over/underexposure in certain outside conditions
- A lot of image noise in low lighting conditions, e.g., during the night (which the WeathercAIm model seems to confuse with rain/snow, as also a human could).

In addition, the different environmental setting, e.g., camera placement, different urban scenes compared to the used dataset will play a role. Another observation was that special (non-weather) cases are encountered in real life that are not present in the training dataset and need special attention, like tunnels, vehicle in garage, etc. To handle these special cases, in principle an extra task could be added by creating extra classification classes that can be added to the WeathercAIm CNN classifier. To further process these special cases, the rule-based system can be used and updated as they are likely to occur only rarely. These observations confirm the need for (re-)training on actual captured images from the field development phase to improve the WeathercAIm model.

### 3.2. Calibration of Sensor Measurements

**Figure 2:**
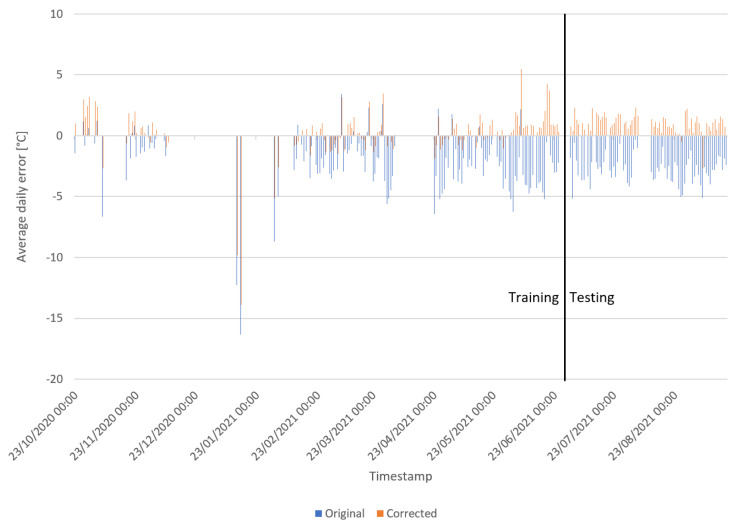
Overview of corrected and original T2M measurements in comparison to the forecasted T2M by INCA-BE.

In our first experiment, we compare the measured T2M to the forecasted T2M by INCA-BE. First, we preprocess the data by splitting the dataset into training and testing with the last 25% as the test set. The data are scaled using MinMax scaling. In total, around 4.5 million samples are used as training data and 1.5 million are used as evaluation. To make our results interpretable, we use the daily average on our metrics. These metrics consist of the mean absolute error, mean squared error, and mean bias. As shown in Table 3, our model can correct the temperature. However, the error on our training set is still significant after training. This can be explained by our use of pseudo labels. The period of the labels is 10 min, while our measurements are on a 3–4 s interval. This difference will result in a fixed error as the real weather changes based on the situation in space (e.g., effects of traffic, sunlight, etc.). As we improved using the correction algorithm, there is still a bias present in the testing set. This might be caused by using the last months as evaluation data. As ambient conditions are seasonally bound, evaluation on a different season can lead to a fixed bias. Figure 2 illustrates the error between our predictions and labels as well as the error between the original measurements and labels. In this figure, we can see that our correction has a positive effect on accuracy. The largest error occurs around 23 January 2021. This can be caused by our sensors that are still operating while the vehicle is for example in a depot. In such a case, the measurements occur indoors while the label is the forecasted temperature outside. A possible solution could be to filter out values when the vehicle is standing still. However, as our fleet consists of postal delivery vehicles, frequent stops occur too much to remove these valuable measurements.

**Figure 3:**
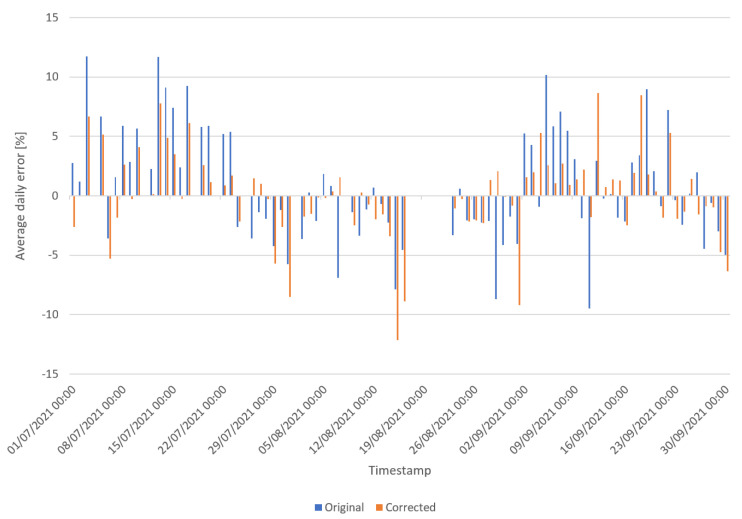
Overview of corrected and original humidity measurements of the testing set in comparison to forecasted humidity by INCA-BE.

In a second experiment, we train the same architecture to correct the humidity measurements. The data used in this experiment include every measurement after July 2021 until September 2021. In July, we made a change to the sensor box, creating ventilation holes to improve the humidity measurements. These data are preprocessed in the same way as the T2M but with random sampling between training and testing with a 25% split as the testing set. We make use of random sampling to have better coverage on our limited dataset. Figure 3 illustrates the error between the corrected signal and our pseudo labels as well as the original measurements and the labels of the testing set. Results are shown in Table 3. Here, we can see the accuracy is improved overall. The difference between training and testing is very small, meaning our model has a good generalisation.

### 3.3. Road Weather Model

**Figure 4:**
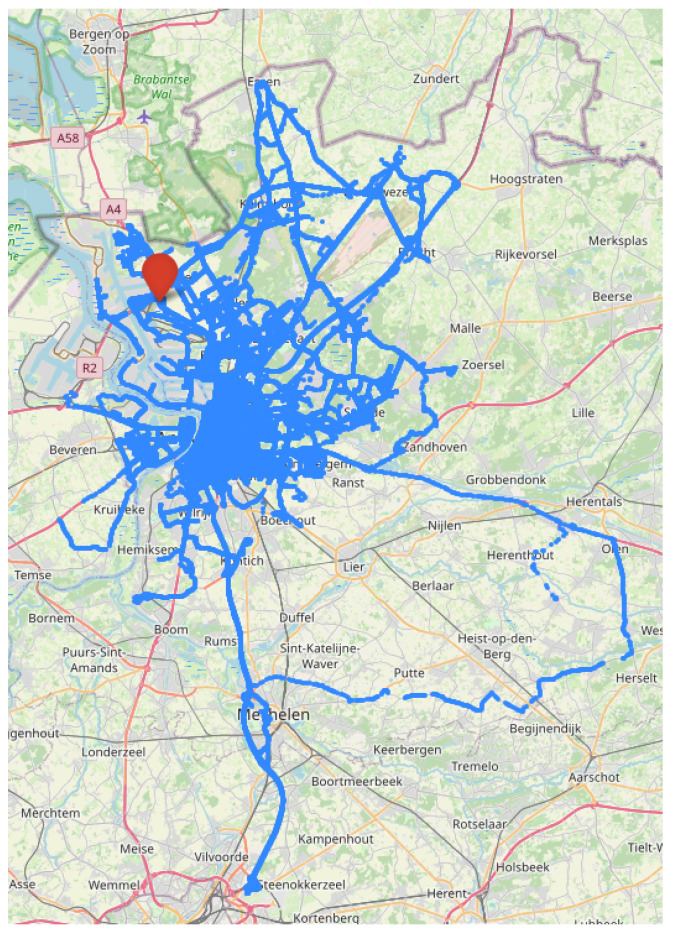
Blue road segments have observations between 19 December 2020 and 1 December 2021. Stabroek RWS is highlighted with a marker.base map and data from OpenStreetMap and the OpenStreetMap Foundation.

The SARWS data, aggregated by BeMobile for each road segment, are displayed in Figure 4. This figure clearly shows that various types of roads have been covered by the cars of the project, which means that weather information from several types of environments were collected, e.g., on highways and in the city center.

**Figure 5:**
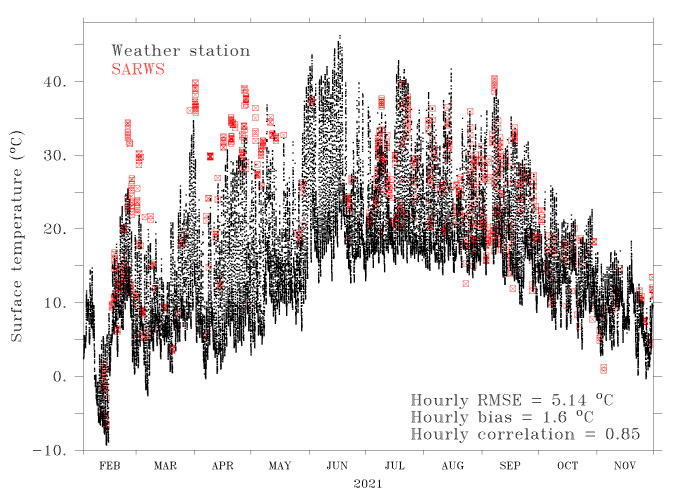

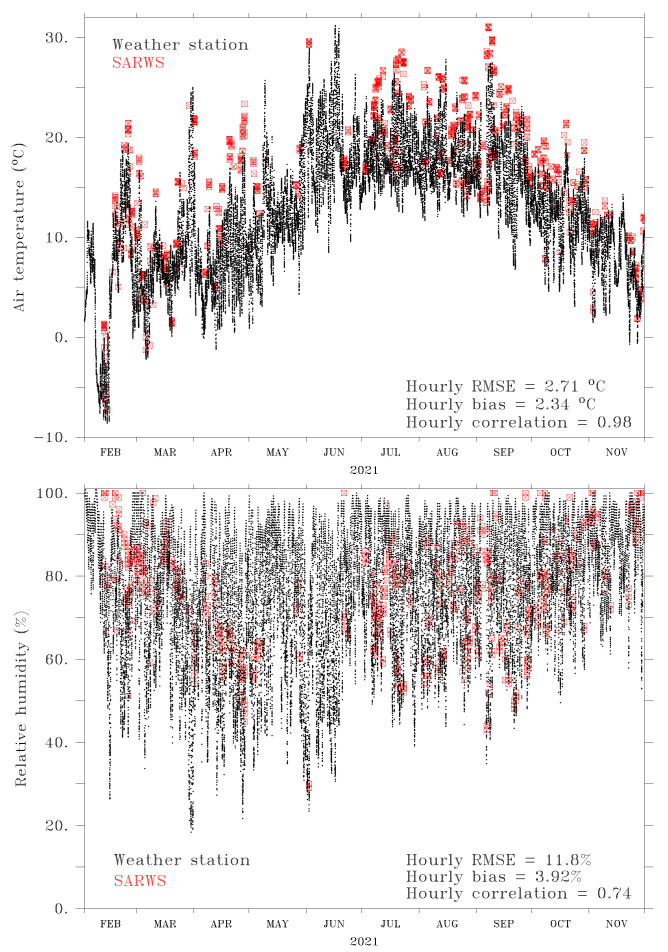
RST (**top**), T2M (**middle**), and RH (**bottom**) between 1 February 2021 and 1 December 2021 at the RWS of Stabroek (black) and from the Bpost cars (red) for nearby road segments (distance to the RWS of maximum 500 m). The scores include the RMSE, bias, and correlation, which are computed from the times series binned on an hourly basis. RST values below −30 ${}^{\circ }$C are considered unrealistic and not used in the computation of the scores.

To assess the quality of the aggregated SARWS data, we compared these with observations from the RWS, an Automatic Weather Station (AWS), the Vlinder network [46], VMM (Flanders Environment Agency) stations, and SYNOP stations that are close enough to the itineraries, that is, less than 500 m. The AWS never meets this criterion and thus is excluded from the comparisons. Figure 5 shows an example of this comparison for the RWS of Stabroek and the following variables: RST, T2M, and RH. Verification for this RWS was first performed in [43] for a shorter verification period. Until 11 March 2021, the SARWS T2M and RH can originate from the Bpost cars or the INCA outputs. The SARWS and the RWS data show a high correlation (>0.8 for RST, >0.9 for T2M, >0.7 for RH), although there is a positive bias of 1.6 °C for RST, 2.3 °C for T2M, and 3.9% for RH. The RMSE is 5.1 °C for RST, 2.7 °C for T2M, and 11.8% for RH. The correlation (r) between SARWS data and observations from other weather stations is also generally high (time period: 19 December 2020–1 December 2021, not shown): 12 stations out of 14 with a r > 0.7 for RH, six stations out of six with a r > 0.9 for T2M. Note that the T2M of these comparisons is not necessarily observed exactly at 2 m.

**Figure 6:**
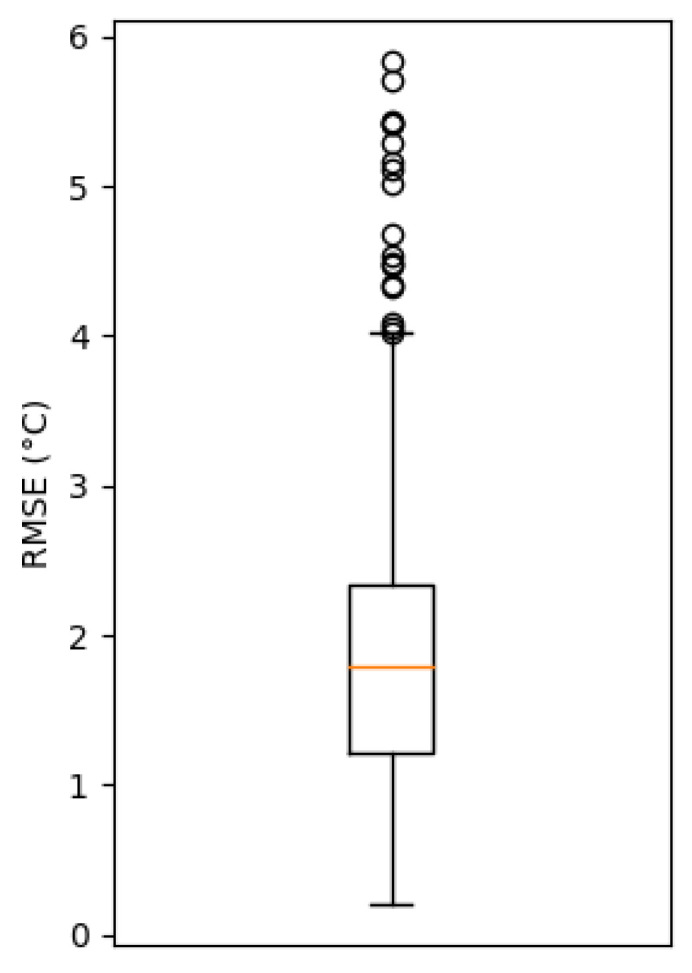
Box and whisker plot of the RMSE between the RWM_SARWS forecasts of RST at the RWS of Stabroek and at close observed road segments (<=500 m) computed for the whole forecast length (2 h). The RMSE box extends from the first to the last quartile, while the orange line corresponds to the median. Each whisker corresponds to a length of maximum 1.5 interquartile range. The time period ranges from 6 May 2021 to 1 December 2021.

To assess the impact of car sensor observation integration into the RWM, we performed a first validation of RWM_SARWS for the time period 6 May 2021 (the data assimilation was corrected on this date) to 1 December 2021. RWM_SARWS forecasts for each road segment are performed every 20 min from H + 0 to H + 2 with a time step of 10 min. These forecasts are compared to the ones performed at the RWS of Stabroek (with RWS observation integration) when the distance between the two forecast locations is less than or equal to 500 m. In Figure 6, we show the RMSE computed for the RST forecasts between H + 0 and H + 2, for the time period between 6 May 2021 and 1 December 2021. The median RMSE is below 2 °C.

## 4. Discussion

In this paper, we presented our research on the use of car fleet sensor data towards real-time road weather warnings and a higher-resolution road weather model. We demonstrated two examples of how AI/ML can be leveraged to process data from a fleet of vehicles and obtain relevant weather information with an improved data quality compared to the raw measurements. First, we showed how convolutional neural networks can be used to extract relevant weather information (visibility and precipitation) from raw camera images. Second, we demonstrated how neural networks can be used to calibrate sensor measurements to reduce the observation errors and improve the data quality.

An updated version of the road weather model, denoted RWM_SARWS, was presented that was specifically developed at RMI to enrich the modeling of road weather with the cars data at a high spatio-temporal resolution. The preliminary results show a median RMSE below 2 °C between the RST forecasts performed at a RWS and the ones performed at close observed road segments. This could be partly explained by the errors from the cars’ sensors. Further improvement is expected in the future, when the demonstrated ML-based calibration method is used operationally. The extracted information from wipers and camera images will also be put to use in a next version of RWM_SARWS, which is expected to further increase the accuracy of the forecasts.

## Figures and Tables

**Table 1:** External sensors mounted on the vehicle fleet.

In Car	Sensor Box
GPS-module	Gyroscope
Camera	Accelerometer
Thermal imaging sensor	Temperature sensor
	Humidity sensor

**Table 2:** WeathercAIm validation classification metrics for different training parameters.

Optimiser	Learning Rate	Dropout Rate	Batch Size	Loss	Accuracy	AUC	F1
sgd	$1\times 10^{-6}$	0.5	64	0.838	0.949	0.997	0.988
sgd	$1\times 10^{-6}$	0.5	16	0.866	0.932	0.996	0.97
sgd	$1\times 10^{-7}$	0.2	16	0.89	0.928	0.996	0.98
nadam	$1\times 10^{-6}$	0.6	16	0.921	0.897	0.991	0.992
nadam	$1\times 10^{-6}$	0.7	16	0.907	0.889	0.988	0.988
nadam	$1\times 10^{-6}$	0.8	16	0.921	0.889	0.984	0.969
nadam	$1\times 10^{-6}$	0.9	16	0.952	0.878	0.982	0.962

**Table 3:** Results of corrections on measurements.

	Mean Squared Error		Mean Absolute Error		Mean Bias	
	Train	Test	Train	Test	Train	Test
Temperature original	3.680 ${}^{\circ }$C${}^{2}$	3.360 ${}^{\circ }$C${}^{2}$	2.916 °C	3.023 °C	−2.780 °C	−3.003 °C
Temperature corrected	1.695 °C${}^{2}$	1.705 °C${}^{2}$	1.285 °C	1.409 °C	0.100 °C	1.077 °C
Humidity original	7.248 ${\%}^{2}$	7.259 ${\%}^{2}$	5.718%	5.732%	1.167%	1.176%
Humidity corrected	5.644 ${\%}^{2}$	5.561 ${\%}^{2}$	4.262%	4.265%	0.172%	0.182%

## Data Availability

The AWD dataset used is publicly available [39].

## Abbreviations

The following abbreviations are used in this manuscript:

AI	Artificial Intelligence
AUC	Area Under (the ROC) Curve
AWD	Adverse Weather Dataset
AWS	Automatic Weather Station
CNN	Convolutional Neural Network
CV	Computer Vision
DNN	Deep Neural Network
GDPR	General Data Protection Regulation
GPS	Global Positioning System
ICSSN	In-Car Smart Sensor Node
INCA	Integrated Nowcasting through Comprehensive Analysis
INCA-BE	INCA Belgium
ML	Machine Learning
NWP	Numerical Weather Prediction
RH	Relative humidity
RMI	Royal Meteorological Institute of Belgium
RMSE	Root-Mean-Square Error
ROC	Receiver Operating Characteristic
RWM	Road Weather Model
RWS	Road Weather Station
SARWS	Secure and Accurate Road Weather Services
SGD	Stochastic Gradient Descent
T2M	Air temperature at 2 m
TD2M	Dewpoint temperature at 2 m
RST	Road surface temperature
VMM	De Vlaamse Milieumaatschappij
ZAMG	Zentralanstalt für Meteorologie und Geodynamik
